# A Lesson for the Future; Determining the Prognosis of the Pregnant Patients with COVID-19 in the Second Trimester? A Case Report

**DOI:** 10.22088/cjim.13.0.284

**Published:** 2022

**Authors:** Maryam Dehghan, Neda Ebrahimian, Leila Mousavi Seresht, Mahtab Ebrahim Babaei

**Affiliations:** 1 Department of Obstetrics and gynecology, Isfahan Medical Science School, Isfahan, Iran

**Keywords:** COVID-19, pregnancy, Remdesivir, prognostic factor.

## Abstract

**Background:**

Coronavirus disease 2019 (COVID-19), the third coronavirus epidemic outbreak, has brought about a lot of concern about the pregnancy and the disease course, fatality rate, and the best management in severe cases. Early April 27, 2020, the first maternal mortality due to progressive COVID-19 infection was reported. Considering this challenging situation, the need for some comprehensive data on the main risk and predictive factors of disease progression is clear.

**Case Presentation:**

Here, we present our experience with 4 confirmed pregnant cases of COVID-19 in the second trimester, who showed typical COVID-19 symptoms like fever, cough, and myalgia. We aim to compare our findings with prior reports by reviewing the most recent and relevant studies.4 cases of COVID-19 induced respiratory discomfort in the second trimester of pregnancy were admitted to the intensive care unit. Most of the cases showed respiratory failure that led to intubation, but despite the similar initial presentation, they revealed widely. All of the required medical records are described here.

**Conclusion:**

Considering the limited information on this new COVID-19 clinical courses in pregnancy in comparison with two prior coronavirus outbreaks since the early 21 century, SARS-Cov and MERS-Cov, the possibility of poor pregnancy outcome has been confirmed, but there is debate on the effect of pregnancy and different management on disease progression. Taking into account the avail of this finding, our experiences, against prior belief, suggest pregnant patients are susceptible to severe morbidity and mortality, similar to report on pregnancies with SARS and MERS.

Recent coronavirus pandemic as the deadliest epidemic in this century, announced as the world emergency with more than 30 million affected people (1, 2). High mortality ratio of prior coronavirus infection in pregnancy, makes it a matter of concern (3-5). Based on our knowledge and experience, it sounds gestational age and ethnicity might have key role in obstetric outcome (5-14). In order to provide a better prospective, here we have addressed some high decompensating velocity patients in the second trimester of pregnancy. Four critical COVID-19 cases in the second trimester have been admitted in a level 3 maternity hospital in Iran, from April to mid-July, 2020. They have been approached by multidisciplinary team consisting of prinatologist, infection-internist and anesthesiologist.

## Case Presentation

The diagnostic criteria of COVID-19 was based on reverse transcription polymerase chain reaction (RT-PCR) on nasopharyngeal and oropharyngeal specimen and presence of ground-glass-opacities in chest-CT scan, though, one was confirmed with histologic investigation consequently after death. The ethical committee’s rules are considered in this report (Number: 199143).Despite similar presentation, 3-day-lasting fever and respiratory discomfort, tachycardia (between 120-140beat/min) and normal peripheral oxygen saturation (O_2_Sat) in all, and same initial medication (Meropenem, vancomycin, hydroxychloroquine and Atazanavir, except case one), there were double maternal and perinatal mortality, and conversely double fortunate outcome. Their Demographic finding and clinical courses are assorted below and in the form of tables 1and 2.

**Table1:** The demonstration of demographic and clinical characters of the infected cases

**Characteristics and outcomes**	**Case 1**	**Case 2**	**Case3**	**Case 4**
Maternal age (y)	26	21	21	36
Gravida, para	G1	G2P1	G1	G2P1
Gestational age on admission (weeks)	17	21	24 (undelivered)	25 (undelivered)
Comorbidities	Gestational Diabetes	No	No	NO
BMI	20	21.5	23	22.3
Blood type (Rh)	O+	AB+	AB+	O+
Fever	Yes	Yes	Yes	Yes
Cough	Yes	Yes	Yes	Yes
Dyspnea	Yes	Yes	Yes	Yes
Myalgia	No	Yes	Yes	No
Headache	No	No	Yes	No
Nausea	No	No	Yes	No
Close contact	No	No	No	yes
Antivirals	Yes	Yes	Yes	yes
Antibiotics	Yes	Yes	Yes	yes
Anticoagulants	No	Not continued	Yes(prophylactic)	Yes(therapeutic)

BMI, Body Mass Index

**Table 2:** The comparison between paraclinical character of infected cases, in demonstrating the most prognostic factor in maternal and prenatal outcome in COVID-19-pregnant cases

Characteristics and outcomes	Case 1	Case 2	Case3	Case 4
SARS-CoV-2 NAAT	Negative	Positive	Positive	Positive
Hemoglobin (g/dL)	6.2-10.8	7.3-11	8.4-10.5	9.6-12.5
Platelets (× 10^3^/μL)	15000-108000	33000-71000	113000-288000	201000-585000
Leukocytes (× 10 /L)	4800-39700	3900-13200	5600-9000	5400-17800
Lymphocyte (% 10^9^/L)	6.2-10	3.4-25	6-22.6	8.7-20
CRP (mg/L)	55-85	27-110	66-82	6-150
AST (U/L)	482-13000	26-3126	20-28	22-85
ALT (U/L)	476-7500	13-1500	11-14	21-100
LDH (U/L)	662-13577	705-4061	560-232	399-772
Creatinine, (mg/dL)	1.5-2.9	0.8-4.4	0.5-0.7	0.6-0.9
Procalcitonin, (µg/l)	NA	>10	1.2	0.4
D dimer (µg /ml)	>15	>10	0.45	0.3
ferritin(ng/ml)	>1645	>1650	71	NA
Cardiac troponin(ng/ml)	2.36	<0.02	2.5	0.2
PT, (seconds)	13-39	11.9-21	12-12.9	10.5-11.4
PTT, (seconds)	31-69	34-68	30-41	35-47.6
INR	1.1-3.7	1.08-2	1-1.2	1-1.01
Admission period(day)	3	30	7	10
Out come	Death	Death	Discharged	Discharged

NAAT, nucleic acid amplification tests; CRP, C-Reactive Protein; AST, aspartate aminotransaminase; ALT, alanine aminotransferase; LDH, Lactate dehydrogenase; PT, prothrombin time; PTT, partial thromboplastin time; INR, international normalized ratio

**Case 1: **A 26-year-old gravida1 woman with history of gestational diabetes and smoking had been admitted at the 17^th^ weeks of gestation (GW) with complaint of vaginal leak, above the mentioned symptom, which had ended-up in spontaneous abortion, exactly at admission. With regard of intensive care, her condition deteriorated rapidly on the same day; septic shock, academia, gastrointestinal bleeding, and need for intubation occurred. In the next 24-hour, multiorgan failure in figure of disseminated intravascular coagulation, pancytopenia, liver and renal dysfunction, and even heart failure also developed. At last, cardiopulmonary arrest happened only after 2 days of supportive care. Although the initial nasopharyngeal RT-PCR was negative, the suspected COVID-19 on chest-X-ray was confirmed by cytopathic change in histologic examination of lung samples, with no sign of infection in uterine tissue. 

***Case 2:*** A 21-year-old previously healthy gravida, at 21^th^ GW was admitted with pancytopenia, liver dysfunction and coagulopathy; anasarca edema, and multi-organ failure emerged within 24-hour. In this meantime, she experienced spontaneous uterine contraction and delivery of non-viable fetus. Despite initiation of hemodialysis, heart failure occurred, which dramaticallyreversed after 7-course of plasmapheresis. Unfortunately acquired-bacterial pneumonia with Acinetobacter in regard of Tazocin, Colistin and Linezolid prescription deteriorated her cardiopulmonary status and she expired on day 28.

***Case 3:*** A 21-year-old previously healthy gravida 1 at 24^th^ GW had been admitted with similar symptoms. Although significant hypotension and pancytopenia occurred within a few hours, fortunately, she recovered over the following 72 hours, only with addition of prophylactic unfractuated-Heparin and successfully discharged. At the time of this report she is in her 37^th^ GW.

**Case 4:**A 36-year-old gravida referred to the hospital at 25^th^ GW, due to rapid deterioration of respiratory condition. To cure, 120mg/day methyl-prednisolone was prescribed, but it did not sound to be helpful, and respiratory-rate raised up-to 54/Min, PAP was in about 50mmHg and O_2_Sat decreased to 81%, even with nasal oxygen, which made us add Remdesivir and therapeutic anti-coagulant. Successfully, 6-day later, oxygen requirement and PAP gradually improved and she was able to discharge with order of corticosteroid tapering. Now she is under antenatal care, in her 35^th^ GW.

**Figure:**
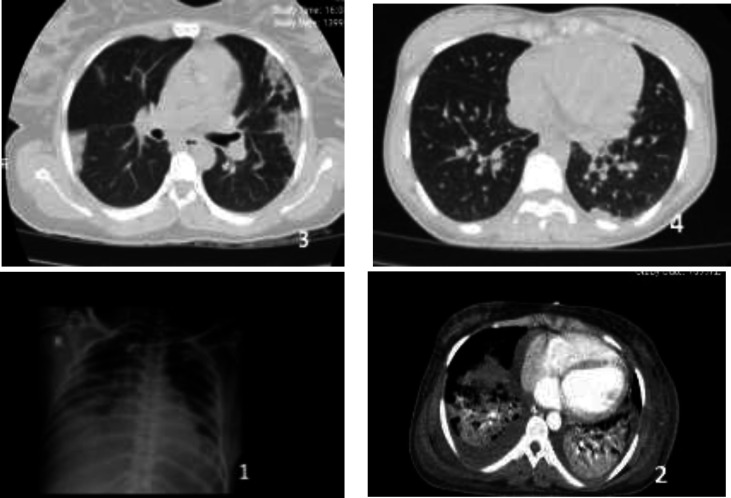
Imaging findings illuminated, ground-glass opacities in lung (each picture are numbered as the cases, respectively)

## Discussion

Pregnancy, contrary to prior belief, could be a major risk factor in COVID-19 severity (Zaigham& Andersson, 2020). Considering, over activation of immune system as the underlying cause of severe feature in COVID-19 (Zaigham& Andersson, 2020, Sun et al., 2020), what is the main explanation for maternal outcome, when physiologic-altered immune system is expected? The other issue is prenatal outcome due to susceptibility of placenta for hypoxia, thrombosis and abruption, with the same pathology as respiratory impairment (6, 9). The next challenge in pregnancy as a critical period of life is considering the risk of management-delay due to high-false negative rate of RT-PCR and the non-characteristic feature of COVID-19 in pregnant’ chest-Ct-scan, as we have experienced parenchymal ground glass opacities instead of expected-bilateral consolidation (6, 12, 13). Contrary to prior report, in our report, obesity did not have an adverse effect, and eradicating the superimposed-bacterial infection or even pregnancy termination does not act beneficially; conversely, it seems spontaneous abortion is an obvious sign of diseaseseverity (5, 14, 15). Furthermore, another great task is objecting the value of lymphopenia, elevated lactate dehydrogenase and procalcitonin level (PCT) in precisely predicting the prognosis (6, 10, 13). Instead, we have confirmed, as indicated in table, the correlation of coagulopathy, D-dimer and so microvascular thrombosis with the intensity of inflammatory storm and hypoxic-related-endothelial cell damage, and recommend these markers in disease monitoring (16, 17). Considering our experience, in spite of previous underestimated dimension of thromboembolic risk in Asian population, we emphasize the necessity of prescribing anti-coagulant agent in order to inhibit thrombin generation, in pregnant patients (9, 18-20). Moreover, it seems that ferritin, creatinine and cardiac troponin, with contrary effect in our cases, have the capability of illuminating the inflammatory response state, as a leader for anti-inflammatory treatment planning, consisting of plasma-exchange or glucocorticoid and Remdesivir in our cases, although their worth is on debate, yet (21, 22).

In conclusion, considering COVID-19 hazardous effects in early gestational age, as have been demonstrated in the present study, and regarding the scarcity of prior studies on the effect of COVID-19 in the gestational periods (Rodrigues et al., 2020), we must emphasize the value of prompt management in pregnancy, especially in early gestational period; on the other hand, we must take into account the rule of coagulopathy-state and inflammatory-induced markers on timely decision-making in eliminating the maternal mortality.
